# Mass and charge distributions of amyloid fibers involved in neurodegenerative diseases: mapping heterogeneity and polymorphism†
†Electronic supplementary information (ESI) available: Experimental section and supplementary figures. See DOI: 10.1039/c7sc04542e


**DOI:** 10.1039/c7sc04542e

**Published:** 2018-02-05

**Authors:** Jonathan Pansieri, Mohammad A. Halim, Charlotte Vendrely, Mireille Dumoulin, François Legrand, Marcelle Moulin Sallanon, Sabine Chierici, Simona Denti, Xavier Dagany, Philippe Dugourd, Christel Marquette, Rodolphe Antoine, Vincent Forge

**Affiliations:** a Univ. Grenoble Alpes , CNRS , CEA , BIG/CBM/AFFOND , F-38000 Grenoble , France . Email: vincent.forge@cea.fr; b Institut Lumière Matière , UMR 5306 , Université Claude Bernard Lyon 1 , CNRS , F-69622 Lyon , France . Email: rodolphe.antoine@univ-lyon1.fr; c ERRMECe , I-MAT FD4122 , Université de Cergy-Pontoise , F-95302 Cergy-Pontoise Cedex , France; d Enzymology and Protein Folding , Centre for Protein Engineering , InBIOS , University of Liège , 4000 Liège 1 , Belgium; e Centre de Recherches des Instituts Groupés , Haute Ecole Libre Mosane , Mont Saint-Martin, 41 , 4000 Liège , Belgium; f Radiopharmaceutique Biocliniques (INSERM U1039) , Faculté de Médecine de Grenoble , F-38700 La Tronche , France; g Département de Chimie Moléculaire , Univ. Grenoble Alpes , CNRS , UMR 5250 , F-38000 Grenoble , France

## Abstract

Heterogeneity and polymorphism are generic features of amyloid fibers with some important effects on the related disease development. We report here the characterization, by charge detection mass spectrometry, of amyloid fibers made of three polypeptides involved in neurodegenerative diseases: Aβ_1–42_ peptide, tau and α-synuclein. Beside the mass of individual fibers, this technique enables to characterize the heterogeneity and the polymorphism of the population. In the case of Aβ_1–42_ peptide and tau protein, several coexisting species could be distinguished and characterized. In the case of α-synuclein, we show how the polymorphism affects the mass and charge distributions.

## Abstract 2

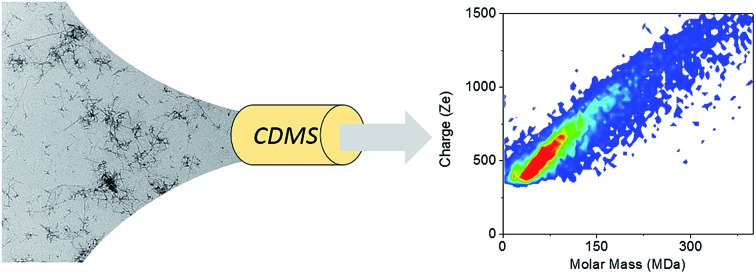
Characterization by charge detection mass spectrometry of amyloid fibers involved in neurodegenerative diseases: Aβ peptide, tau and α-synuclein.

## Introduction

The most frequent age-related neurodegenerative diseases, Alzheimer's and Parkinson's diseases, are related to the accumulation of amyloid deposits due to the aggregation of specific proteins.^1^,^2^ In the case of Alzheimer's disease, Aβ peptides form extracellular plaques and tau protein accumulates as intraneuronal inclusion bodies.^3^,^4^ This is observed in relation to synaptic dysfunction, neuron death, brain shrinkage and, ultimately, dementia. Parkinson's disease is associated to the appearance of intracellular deposits made of α-synuclein, so-called Lewy bodies, leading to dopaminergic neuron death (lack of DOPA synthesis) and to motor system disorder.^5^ The proteins involved in these deposits are in the so-called amyloid state, with common structural features:^6^,^7^ high aspect-ratio fibers, with diameters of a few nanometers and lengths around a micrometer, stabilized by hydrogen-bonded β-strands perpendicular to the fiber axis and forming β-sheets. Beyond these generic features, amyloid fibers are characterized by a polymorphism which is observed within *in vivo* amyloid deposits^8^,^9^ and within samples prepared *in vitro*.^10^–^12^ As amyloid fibers are often an association of protofibrils, their heterogeneity, *i.e.* their polymorphism, depends on the number of protofibrils, the arrangement of protofibrils or the conformation of polypeptide.^10^

According to the classical view, the formation of amyloid fibers follows a nucleation/growth mechanism, *i.e.* a primary nucleation mechanism.^13^ The initial step, which is also the slowest one, is the formation of oligomers which act as nuclei for the growth of the fibers. In the case of Aβ_1–42_ peptide, these oligomers can be of various sizes from dimers up to dodecamers.^14^–^16^ Moreover, preformed fibers potentially enable an additional nucleation pathway, so-called secondary nucleation mechanism.^17^–^19^ Their surface can act as template for the formation of oligomers and protofibrils, and their fragmentation can generate new growth sites. At high ratio of preformed fibers, the fibril-dependent secondary nucleation mechanism can surpass the primary nucleation and becomes the main source of new nuclei.^19^–^21^ This could provide a relationship between the accumulation of amyloid deposits and toxicity *in vivo*,^19^ through the constant release of oligomers, thought to be the most toxic species.^22^–^26^

We have shown recently that charge detection mass spectrometry (CDMS) can be used to accurately measure masses of individual amyloid fibrils,^27^ while previous MS-based studies of fibrillation have been limited to the early steps in aggregation.^28^,^29^ The mass of the biological assembles of few megadaltons to 18 MDa can be measured by native mass spectrometry^30^–^34^ but it is very challenging due to the charge states resolving problems.^35^,^36^ Single-molecule CDMS technique, where mass and charge are measured simultaneously, has previously been used for DNA, polymers and various virus capsids.^37^–^45^ In our previous study, samples containing a single population of amyloid fibrils have been characterized.^27^ This provided important information about amyloid fibrils, such as their mass, charge density and the number of proteins involved. However, disease-related amyloid fibrils are also characterized by significant heterogeneity and polymorphism;^8^–^10^ mass and charge distribution of large heterogeneous and polymorph amyloid fibrils have never been characterized by any methods. We report here the characterization by CDMS of amyloid fibers made of the proteins involved in neurodegenerative diseases: Aβ_1–42_ peptide, tau and α-synuclein. Beside the mass distribution for the different amyloid fibers, this technique allows to highlight and characterize the heterogeneity of the populations, with the possibility to distinguish several species, as illustrated with Aβ_1–42_ peptide and tau, and to quantify the polymorphism, as illustrated with α-synuclein. In that case, we show how the polymorphism affects the mass and charge distributions.

## Results and discussion

**Fig. 1:**
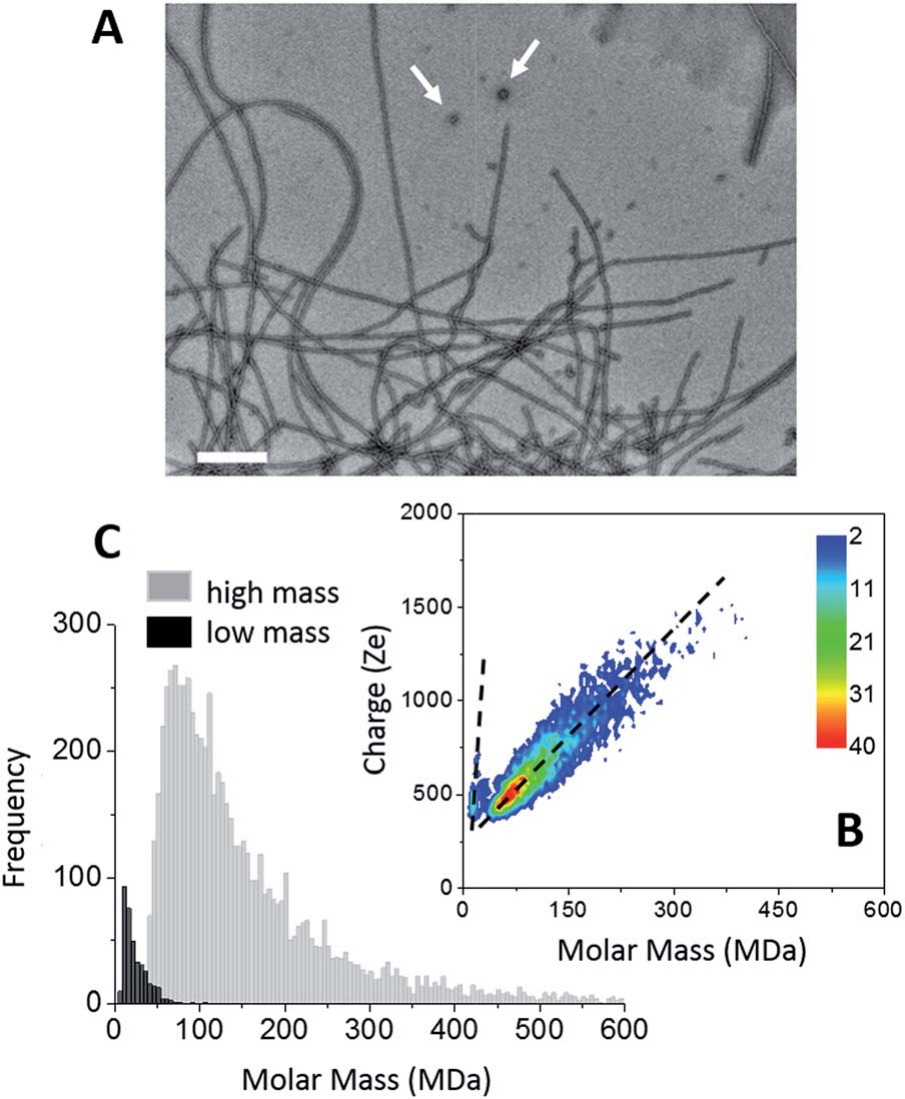
Tau amyloid fibers. (A) TEM image of tau amyloid fibers, scale bar: 200 nm. Typical spherical oligomers are shown with white arrows. (B) 2D-graph of CD-MS measurements performed on tau amyloid fiber sample. (C) Mass distribution drawn from (B). The two populations (high mass and low mass) have been distinguished thanks to their different time of flight. The mass distributions are histogrammed using a given bin-size (5 MDa). Each bar represents the number of measured ions whose masses correspond to the mass range of the bin.

The formation of amyloid fibers by tau was triggered by the addition of heparin at a molar ratio of 2.2 (see “Methods” within ESI†).^46^ Transmission electron microscopy (TEM) of this sample showed that the main species corresponded to well-defined straight fibers together with few more-or-less spherical oligomers (Fig. 1A). Two populations could be seen also on the CDMS 2D-graph (Fig. 1B). They could be further distinguished by their time-of-flight (Fig. 1C and S1†). The main population, the “high” mass population, had a mean mass of 113.5 MDa (Fig. 1C). Because of the heparin, only an estimation of the number of proteins per fibril could be given. Assuming that the ratio tau/heparin was the same within the fibers and in the bulk, *i.e.* 2.2, we obtained around 1835 proteins per fiber on average (*M*_tau_ = 45.85 kDa and *M*_heparin_ = 8 kDa). The length distribution estimated from electron microscopy image was quite broad (Fig. S1†) and this impeded to estimate a mass per length value with some meaning.

The “low” mass population, on the bottom detection limit of the CDMS experiment (around 13 MDa), was probably due to the spherical oligomers seen in the electron microscopy image (Fig. 1A). Based on a protein density at 1.41 g cm^–3^ (ref. 47), a mean molar mass of 13 MDa corresponded to spherical oligomers of about 30 nm, in agreement with the size of the oligomers observed by electron microscopy. The differences of the charge *vs.* molar mass slopes corresponding to the two populations (Fig. 1B) indicated that the oligomers were highly charged; much more than the fibers. The binding of heparin was necessary for the formation of tau fibers in order to screen the charges within the fuzzy coat of the fibers.^46^,^48^,^49^ A possible explanation for the difference of charge density between the spherical oligomers and the fibers was that no heparin was bound to the oligomers. Then, these later contained 283 proteins. Moreover, based on number of macro-ions counted for each population; *i.e.* 364 *vs.* 6836 for oligomers and fibrils respectively, the former represented 5% of the detected macro-ions, and the later 95% of them (Fig. 1C).

**Fig. 2:**
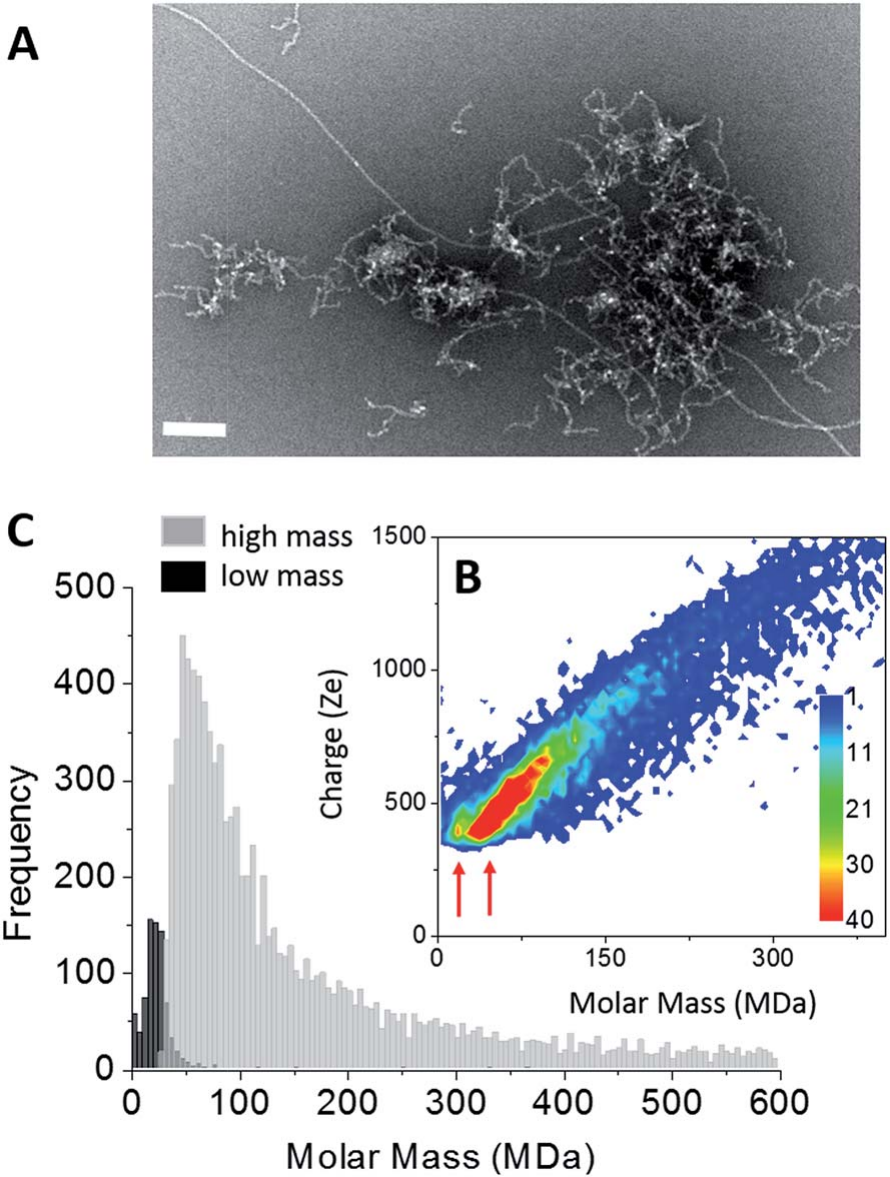
Aβ_1–42_ amyloid fibers. (A) TEM image of Aβ_1–42_ amyloid fibers, scale bar: 100 nm. (B) 2D-graph of CD-MS measurements performed on Aβ_1–42_ amyloid fiber sample. Red arrows indicate the “low” and “high” mass populations. (C) Mass distribution drawn from (B). The two populations (high mass and low mass) have been distinguished thanks to their different time of flight. The mass distributions are histogrammed using a given bin-size (5 MDa). Each bar represents the number of measured ions whose masses correspond to the mass range of the bin.

Amyloid fibers made of Aβ_1–42_ peptides were obtained upon incubation at pH 6.5 and 37 °C. At least two fiber populations: short and curly protofibrils with a strong tendency to aggregate into clusters and elongated straight fibers, could be distinguished on the electron microscopy image (Fig. 2A). From TEM images, the predominant population was attributed to clusters of protofibrils (Fig. 2A and S2†). On a higher magnification image (Fig. S2†), we could see that our sample showed strong similarities with observations reported earlier;^50^ branching on the fiber sides (Fig. S2,† arrows) showed that some secondary nucleation was occurring. Therefore, the final state of our sample was the result of a competition between primary and secondary nucleation mechanisms. Mass measurements have been performed on 9642 single Aβ fiber macro-ions and the results were gathered into a 2D graph (charge *vs.* mass) (Fig. 2B). Although less evident than in the case of tau, two different charge *vs.* mass dependencies could be seen on the 2D graph. Then, based on their time-of-flight, two populations could be extracted from the 2D-graph: a “low” mass (centered on 20 MDa) and a “high” mass population (centered on 55 MDa) (Fig. 2C). We have shown with nanoparticles that CD-MS allowed to distinguish different types of clusters and provided estimations of their relative populations in agreement with TEM measurements.^51^ According to the amount of macro-ions counted for each population: 759 for “low” mass *vs.* 9642 for “high” mass, the “low” mass population counted for 8% of the macro-ions. Based on the obvious ratio of populations within the electron microscopy images (Fig. 2A and S2†), the “low” mass population could be assigned to the elongated fibers, and the “high” mass population to protofibril clusters.

For the elongated fibers, a molar mass of 20 MDa gave 4400 peptides per fiber (*M*_Aβ_ = 4.51 kDa). Their length distribution was centered on 0.9 μm (Fig. S2†), this gave a mass-per-length (MPL) value around 22 kDa nm^–1^, in agreement with the values based on electron cryomicroscopy image processing, *i.e.* ∼20 kDa nm^–1^.^52^ These parameters could not be determined in the case of the “high” mass population which corresponded to protofibril clusters. Nevertheless, the fact that their charge density was lower (weaker slope of the charge *vs.* mass dependency) indicated either that they were less electrically charged or that their association induced some charge screening.

**Fig. 3:**
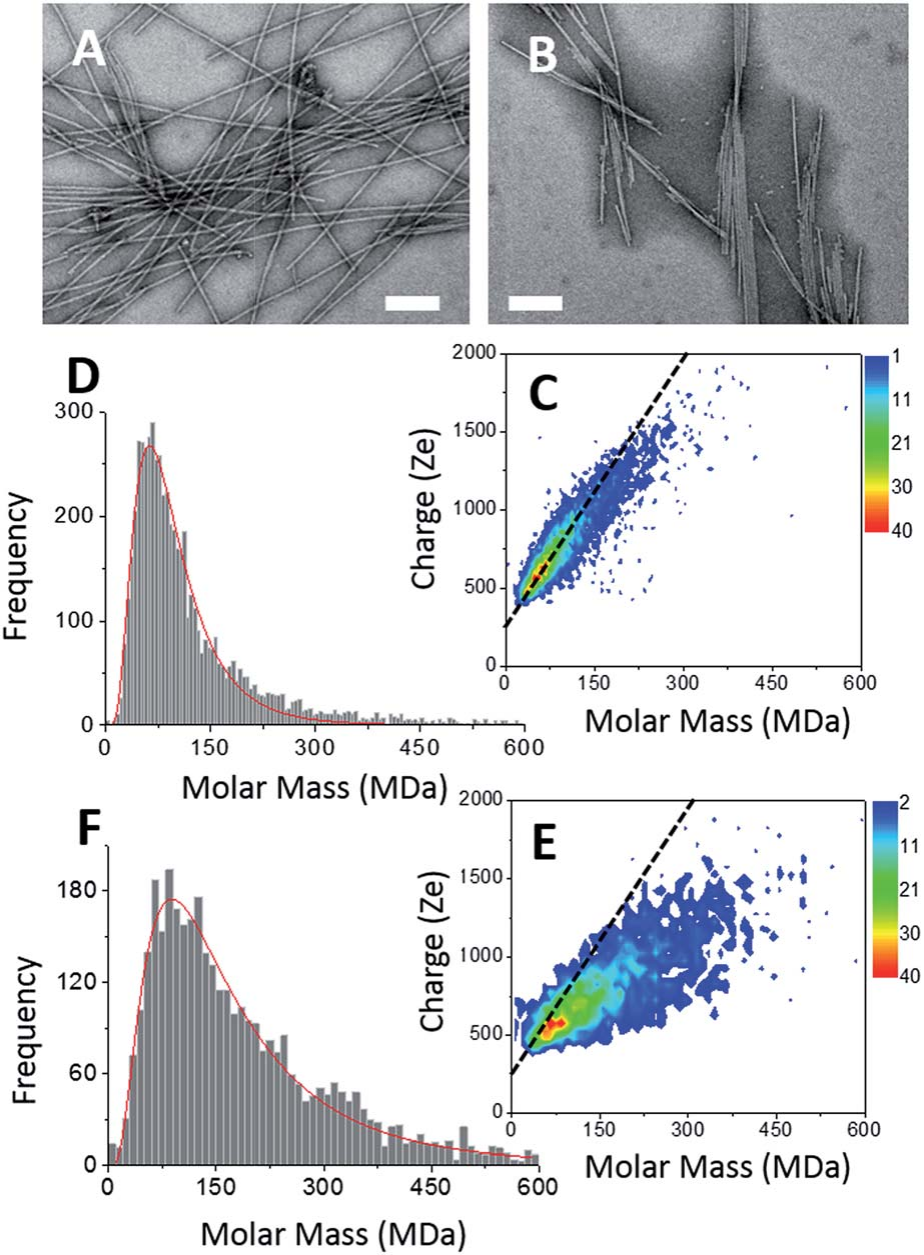
α-Synuclein amyloid fibers. (A & B) TEM image of type I (A) and type II (B) α-synuclein amyloid fibers, scale bar: 200 nm. (C & E) 2D-graph of CD-MS measurements performed on type I (C) and type II (E) α-synuclein amyloid fiber sample. (D & F) Mass distribution drawn from (C & E) for type I (D) and type II (F) α-synuclein amyloid fibers, respectively. The mass distribution is histogrammed using a given bin-size (10 MDa). Each bar represents the number of measured ions whose masses correspond to the mass range of the bin.

Two types of α-synuclein fibers have been obtained. Although the reasons for the differences between the two samples are not fully understood (see ESI†), their characteristic in terms of mass and charge could be clearly discriminated by CD-MS. According to TEM images (Fig. 3A and B), isolated fibers, referred to as type I, were formed in the first sample (Fig. 3A), while irregular ribbons, referred to as type II, were observed in the second sample (Fig. 3B). These ribbons resulted from the heterogeneous association of fibrils of variable lengths. Moreover, fibers involved in ribbons were obviously shorter than those observed in the sample with isolated fibers. According to their respective length distributions extracted from several TEM images (Fig. S3†), isolated fibers of the first sample type had a mean length of 0.9 μm, while those involved within ribbons had a mean length of 0.5 μm. Still according to TEM images, the most populated ribbon species was that made of the association of two fibers (*i.e.* ∼58%), and the probability decreased when the number of associated fibers increased (Fig. S3†). Moreover, the probability to have ribbons made of an even numbers of fibers was much higher than that of ribbons with an odd number of fibrils. Both types of fibers were further characterized by Atomic Force Microscopy (AFM) (Fig. S4†). The height profiles of isolated fibers showed a single maximum around 8 nm. In the case of ribbons, the profiles were much broader, with several peaks corresponding to aligned fibers. At the exception of regions with overlapping fibers, the average height of the ribbons was around 6–8 nm. This was close to the height of isolated fibers suggesting that the ribbons were mostly the results of the lateral association of fibers into 2D structures.

The CDMS 2D graph recorded with the isolated fibers showed a well-defined charge *vs.* mass dependency (Fig. 3C). According to the mass distribution (Fig. 3D), the mean mass was 85.4 MDa. Hence, these α-synuclein amyloid fibers were made of 5900 molecules on average (*M*_α-syn._ = 14.46 kDa). According to the length distribution estimated from electron microscopy (Fig. S3†), the average fiber length was 0.90 μm, giving an estimation of the mean MPL value around 95 kDa nm^–1^, to be compared with that determined from electron microscopy image processing, *i.e.* 60 kDa nm^–1^.^53^ Our value must be taken with caution because of the poor quality of the length distribution extracted from the TEM images (Fig. S3†). Given the disparity in length, a much larger sampling would be required to obtain a precise value.

**Fig. 4:**
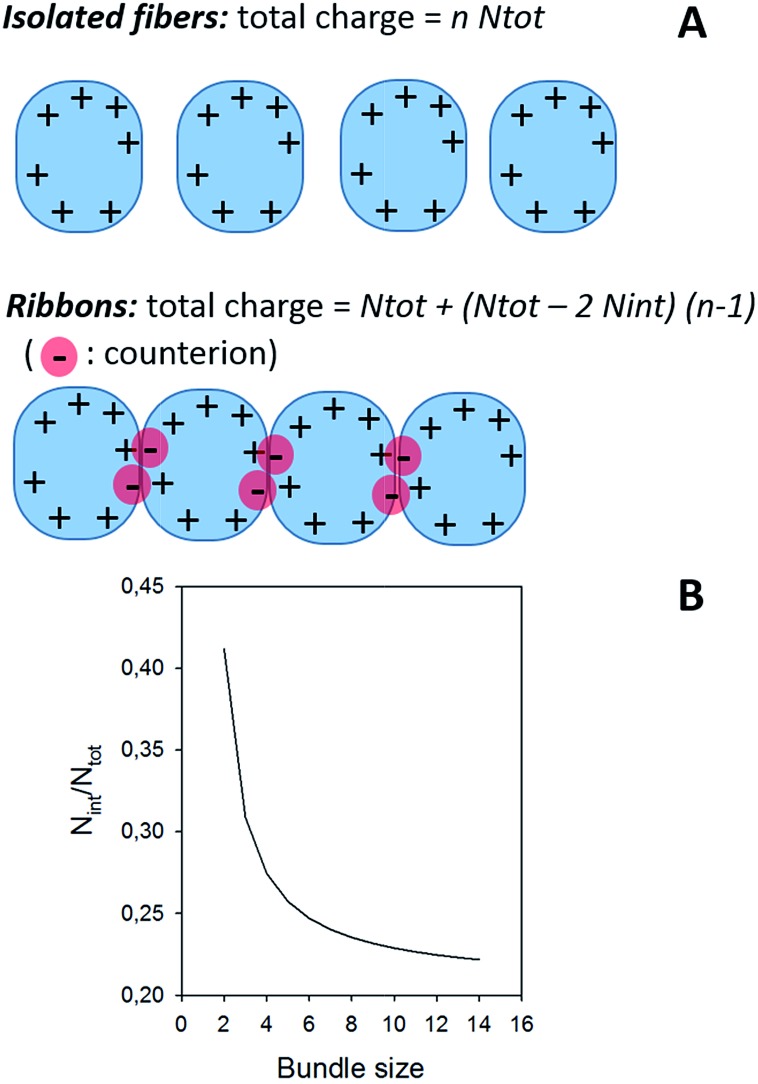
(A) Model for the effect of ribbon formation on charge density. (B) Effect of ribbon formation on charge density. The continuous line corresponds to the evolution of *N*_int_/*N*_tot_ as a function of the bundle size, *i.e.* the number of fibers involved in the ribbon, considering a ratio between the two ‘charge *vs.* mass’ graphs equal to 1.7, which was extracted from Fig. 3. About 20% of the charges are involved in the interaction; the value of *N*_int_/*N*_tot_ tends toward 0.206 for high values of *n*.

In the case of ribbons, the charge *vs.* mass dependency was not so well defined (Fig. 3E), resulting in a much broader mass distribution, with a mean mass at 147.8 MDa (Fig. 3F). The broadness of mass distribution reflected the heterogeneity of the sample, with ribbons of varying lengths and widths. It was obvious on the 2D-graph that the charge of the ribbons was significantly lower than in the case of the isolated fibers; a dashed line corresponding to the charge *vs.* mass dependency of the isolated fibers was reported on both 2D-graphs for comparison. The pH being identical for both types of sample, this suggested that charges were at least partially hidden due to the organization of the fibers into ribbons. In our electrospray ionization (ESI) mass spectrometry (MS) investigation with a positive polarity mode, only positively charged gas-phase electrosprayed fibrils were measured, resulting from a complex desolvation process of highly positively charged solvent droplets. The net charge of electrosprayed fibrils produced by ESI was mainly determined by the number of positively charged sites on their surface. In a previous work,^54^ we demonstrated with latex nanoparticles that the magnitude of charging of ions produced in the gas phase was correlated with the surface charge in solution, however their values cannot be directly compared. Therefore the values of the charge of α-synuclein fibers reported in the 2D graph (Fig. 3C) cannot be compared to those extracted from electrophoretic mobility measurements on fibrils in solution.^55^ However, the phenomena described to explain the fact the fibers in solution are drastically less charged than expected from the charge of monomers, *i.e.* shift of ionizable residue p*K*_a_ values and/or incorporation of counter ions into oligomers,^55^,^56^ must occur in our experiments. Thus, the net charge of electrosprayed amyloid fibers was significantly smaller (more than ten times) than expected from the net charge of monomers monitored by ESI-MS.^27^ According to the electron microscopy and AFM images, the ribbons were due to the 2D association of the fibers. Likely, the weaker charge density of the ribbons was due to the further burying of ionizable groups into the interface, allowed by a shift of their p*K*_a_ values or to the incorporation of counter ions. This allowed to use a simple model assuming some counter ion incorporation (easier to visualize than a shift of p*K*_a_) (Fig. 4) to estimate the effect of the fibrils association on the charge density (charge per mass unit). The ratio between the slopes of the two associated ‘charge *vs.* mass’ graphs, either for single or associated fibrils, is equivalent to the ratio: *r*, between the charges present within a set of either isolated fibrils or laterally associated into ribbons:
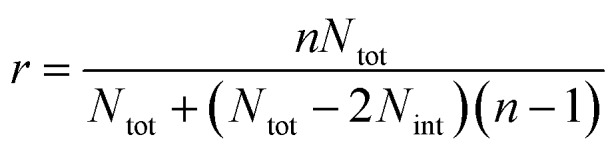
where *n* is the number of considered fibrils, *N*_tot_ is the number of charges carried by a single fibril and *N*_int_ is the number of charges involved in the ribbon association.

Then: 
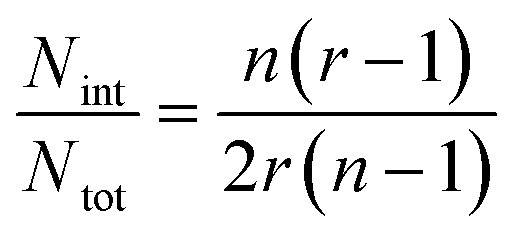
, which tends toward: 
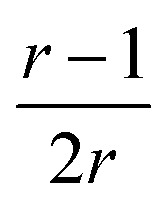
 for *n* ≫ 1. According to this equation and the value of the ratio between the slopes of the ‘charge *vs.* mass’ graphs (Fig. 3C and E), *i.e.* 1.7, about 20% of the charges of fibers are involved in the lateral association.

## Conclusions

Charge-detection-mass-spectrometry provides a wealth of information on amyloid fiber samples. Beside the mass and charge of individual fibers, this technique enables to characterize the heterogeneity of the population and to detect the presence of different types of fibers. This is of prime importance with amyloid fiber samples, well-known to be highly heterogeneous and, as a consequence, difficult to accurately characterize. In association with time-resolved experiment, this will allow to investigate the mechanisms of formation and maturation of amyloid fibers, so important to get insight into the development of the neurodegenerative diseases. The association of classical MS and CDMS with separative methods will allow the complete characterization of the species involved from monomer to amyloid fibers, through oligomers.

## Conflicts of interest

There are no conflicts to declare.

## Supplementary Material

Supplementary information
