# Supplementary material for: Mass and charge distributions of amyloid fibers involved in neurodegenerative diseases: mapping heterogeneity and polymorphism
Source: Chem Sci. 2018 Feb 5;9(10):2791–6. doi: 10.1039/c7sc04542e (PMC5914292; doi:10.1039/c7sc04542e)
Supplement: Supplementary file 1 [file SC-009-C7SC04542E-s001.pdf]

Electronic Supplementary Material (ESI) for Chemical Science.
This journal is © The Royal Society of Chemistry 2018

Methods

Aβ$_{1-42}$* peptide:** Lyophilized Aβ$_{1-42}$ peptide (Genecust, Luxembourg) was dissolved in distilled water at pH 12.0, adjusted with 5 M NaOH. The solution was filtered through a spin 0.2 μm membrane filter (Millipore, n°146560) to remove any aggregated species. The solution was stored at −20 °C. Aβ$_{1-42}$ fibrils were prepared by incubating monomer Aβ$_{1-42}$ peptide solution (final concentration at 100 μM) in Tyrode's buffer (150 mM NaCl, 3 mM KCl, 10 mM HEPES pH 7.4, 10 mM glucose filtrated with a spin 0.2μm) at 310°K in a 500 μL reaction volume on a rotating shaker (300 rpm) at pH 6.5 for 72 hours. The glucose, suitable for other experiments, had no effect on the fibril formation ***(1).

Tau protein: Tau protein was produced using transformed *Escherichia coli* (BL21 DE3) harboring full-length cDNA encoding for htau40 protein isoform (gift of INSERM UMR 788 - CHU de Bicêtre France) following the procedure described by Goedert et al. ***(2)***. After being collected by centrifugation (2500 g for 20 min at 4°C), the cells were suspended in 10 mL of lysis buffer (50 mM PIPES pH 6.8, 50 mM NaCl, 1 mM EGTA, 5 mM DTT, 0.1 mM PMSF) and sonicated on ice (10 times for 20 s, 50 W power). The homogenate was clarified by centrifugation (19000 g for 20 min at 4°C), and the lysate was filtered through successive 0.45 μm and a 20 μm membrane filter before injection on column. Purification of the lysate was performed as described by Csokova et al. ***(3)*** by both cation-exchange and size-exclusion chromatography. Tau containing fractions were pooled, submitted to dialysis against 2x2 L of 10 mM ammonium acetate (6 h at 4°C under stirring) and lyophilized.

Fiber formation: Lyophilized hTau40 was dissolved at 110 μM, in MOPS buffer (3-(N-morpholino)propanesulfonic acid), 20 mM pH 7.4. The fibrils were prepared in MOPS by adding heparin to hTau40 monomers (molar ratio 1:2.2) at 310°K in a 600 μL reaction volume on a rotating shaker (300rpm), pH 7.4 for 72 hours.

Heparin (average molecular mass of 8 kDa), sodium chloride (NaCl, >99.5%), sodium hydroxide (NaOH, 99.99%), 3-(N-morpholino)propanesulfonic acid (MOPS, >99.5%) and 4-(2-hydroxyethyl)-1-piperazineethanesulfonic acid (HEPES, 99.5%), were purchased from Sigma-Aldrich (France). Potassium chloride (KCl, 99.5%) was purchased from SDS Carlo Erba (France).

α-Synuclein: WT human α-synuclein was expressed in *E coli* and purified according to previously published protocol with few modifications ***(4)***. First, *E. coli* cells were lyzed by three passages on a French press instead of by successive freeze-thaw cycles and sonication. Secondly, the ammonium sulphate precipitation step was omitted. Finally, after the anion-exchange chromatography, the protein was further purified by gel filtration using a Sephacryl-

S100HR column and milliQ water was used as running buffer. The protein was lyophilized and stored at -20°C. The integrity of the protein was checked by SDS-PAGE and ESI-MS. The purity of the protein was estimated to be higher than 95% based on the densitometry analysis of the SDS-PAGE.

Fibril formation: Lyophilized protein was dissolved into 3 mL of 10 mM NaP buffer pH 7.5 containing 100 mM NaCl and 0.1% sodium azide. The solution was filtered through a 0.22 µm cut-off syringe filter and the concentration of the protein was determined by absorbance measurement at 280 nm from the extinction coefficient (5960 cm^{-1} M^{-1}). The protein solution (83.4 µM for sample 1 and 51.5 µM for sample 2) was incubated in a 14 mL polypropylene round-bottom corning falcon tube on a New Brunswick Innova 44 incubator at 37°C under orbital agitation at 226 rpm for 12 days. At given time points (roughly every 24 or 48 h), an aliquot was taken to monitor the formation of amyloid fibrils by thioflavin-T fluorescence using previously published protocol ***(5)***. The reasons for the differences between the two samples are not understood. The only notable difference is the initial concentration of monomeric protein: 51 µM in the case of the isolated fibers and 83 µM for the ribbons. As a consequence the fiber formation was faster at higher concentration (data not shown). One possibility is that significant structure changes have been reported during the maturation of α-synuclein fibers, i.e. after their formation. The total incubation time being the same for both samples, the maturation phase must have been longer in the case of the higher protein concentration sample, because of a faster fibrillation, allowing more reorganization ***(6)***.

Transmission electron microscopy: Fibril suspensions were diluted in buffer to around 1 mg/mL concentration and adsorbed into glow-discharged carbon-coated copper grid for 30 seconds. Grids were stained with 2 % uranyl acetate, dried with filter paper and observed with Phillips CM12 transmission electron microscope.

Atomic Force Microscopy: (AFM)**.** Fibrils were diluted to a final concentration of 17 µM in ultrapure 18.2 MΩ water and were allow to adsorb over night at 4°C onto plasma O_2 cleaned glass slides. After removal of the solution, the samples were left to dry. Images were recorded at a scanning speed of 0.4 or 0.5 Hz in the tapping mode in air with a Dimension 3100 (Bruker) using Si_3N_4 cantilevers of a spring constant of 0.4 $N.m^{-1}$, and were analysed using Nanoscope (Bruker).

CDD instrument: In the charge detection device (CDD), the image charge is collected by the "pick-up" tube (high impedance) and is transmitted to a field effect transistor (2SK152). This JFET associated with a preamplifier, converts the current induced voltage. In order to lower the link impedances, the JFET transistor, the preamplifier and the first amplification stage are

mounted on an integrated circuit to the tube. The association with the JFET transistor is provided by a brass screw. And the transmission between the mechanics of the CDD and the PCB is made by a copper rigid connector: in addition to limit ground currents, this rigid link allows to minimize noise. The equivalent capacity of the link between the pick-up tube and the JFET is approximately 1 pF. The entrance and the exit of the pickup tube (stainless tube along 37,5mm and 6 mm in diameter) are capped by tubes of the same inner diameter and 8 mm long. The gap between the end caps and the pick-up tube is 1 mm and allows a fast rise time for the charge image signal.

After the differential amplifier, the resulting output for each ion is a double pulse signal. The first pulse signal is due to the ion entering the detector and the second to the ion exiting the detector. The duration of the signal corresponds to the time of flight (TOF) of the ion through the detector. The TOF of the ion in the 3.75 cm tube is proportional to its m/z value and this gives the ion velocity. The mass m of each macro-ion is obtained from a combination of both z (the charge) and m/z values.

Data processing and analysis: We used a home-developed, VISUAL C++, Windows-based software to record chromatograms. The program calculates the time between the maxima of the positive and negative pulses, the amplitudes of the two pulses and the ratio between their absolute values. A high-frequency filter was added to the data processing of traces in order to remove peak artefacts. Residual droplets are excluded by using post-processing thresholds for TOF (> 80 μs). In this work, only ions (with charges higher than ~300 e) that both enter and exit the tube are counted. Events for which the absolute value of the amplitude ratio between the first and the second pulses is greater than 1.5 or less than 0.75 are automatically excluded. These events may result either from an ion that enters but does not exit the detector (due to fragmentation or loss in the charge detector) or from non-single events including two or more different ions entering the charge detector during a time-of flight measurement. This procedure precludes, in particular, the detection of more than one ion with the charge-detection device. Finally, the corresponding ion counting rate ranges around 100 ions per second.

For each ion, the mass is deduced from its m/z and z values. Each displayed mass distribution compiles about 8,000-10,000 individual ion measurements. The mass distributions are histogrammed using a given bin-size. Each bar represents the number of counted ions having mass corresponding to the mass range of the bar. The shape of the molecular weight distribution (MWD) of fibrils was fitted by a lognormal distribution: $P(\ln M) = \frac{1}{\sqrt{2\pi\sigma}} \exp\left[-\frac{\ln^2(M/m_0)}{2\sigma^2} \right]$,

where m_0 is the median and σ is the width of the distribution. m_{max} is the mass corresponding to the maximum of the lognormal distribution.

ESI conditions: Briefly, buffered aqueous dispersions of fibrils were diluted in a 75/25 water:methanol solvent mixture before injection into the ESI source. Final concentrations range between 25-50 µM in fibrils. Dispersions were injected at flow rates of typically 0.2-0.6 mL/h, and entered the electrospray chamber through a 0.1 mm internal diameter stainless steel capillary tube located inside the needle tip. Nitrogen gas was injected between the end cap and the transfer glass capillary and was flown through a heater typically set at 200 °C. The vacuum interface was composed of a glass transfer capillary that passes the ions into the first stage of the vacuum system, an end cap, a skimmer between the first and second vacuum stages, a hexapole ion guide and an exit lens. The ESI source generates highly charged macro-ions which are guided by an ionic train to the mass spectrometer. Ions are guided up to a vacuum stage chamber (~10^{-6} mbar) and directed through the CDD.

References:

1- Edelman *et al*. (2008) Neurosci. Lett. 441, 319-322
2- Goedert and Jakes (1990) *EMBO J.* **9**, 4255–4230
3- Csokova *et al.* (2004) *Protein Expression and Purification* **35**, 366–372
4- Hoyer *et al.* (2002) *J Mol Biol* **322**, 383-393
5- Scarafone *et al.* (2012) *PLoS One* **7**, e31253
6- Guilliams *et al.* (2013) *J Mol Biol* **425**, 2397-2411

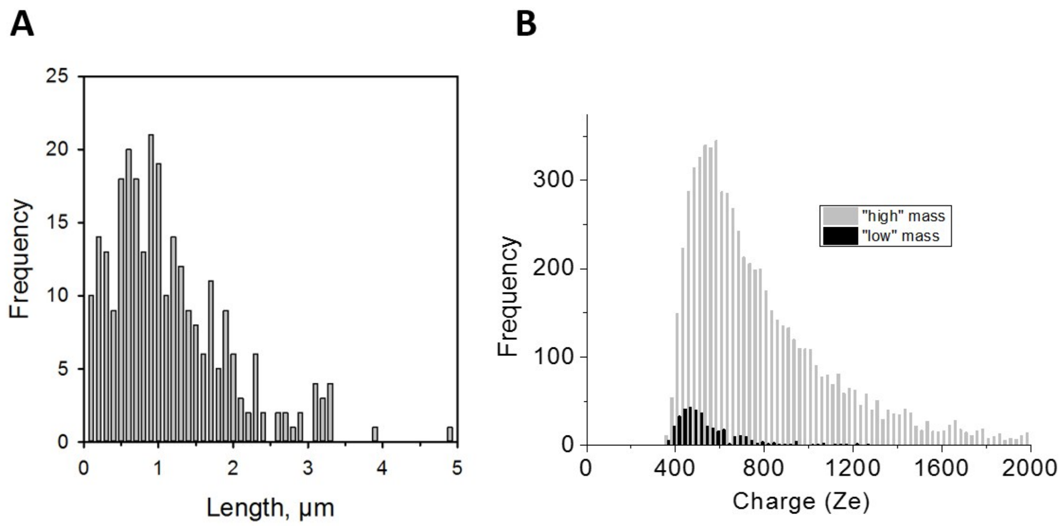

Figure S1: A) Length distribution of Tau amyloid fibers extracted from TEM images. **B)** Charge distribution drawn from Figure 1B. The two populations have been distinguished thanks to their different time of flight. The charge distribution is histogrammed using a given bin-size (25 e). Each bar represents the number of measured ions whose charges correspond to the charge range of the bin.

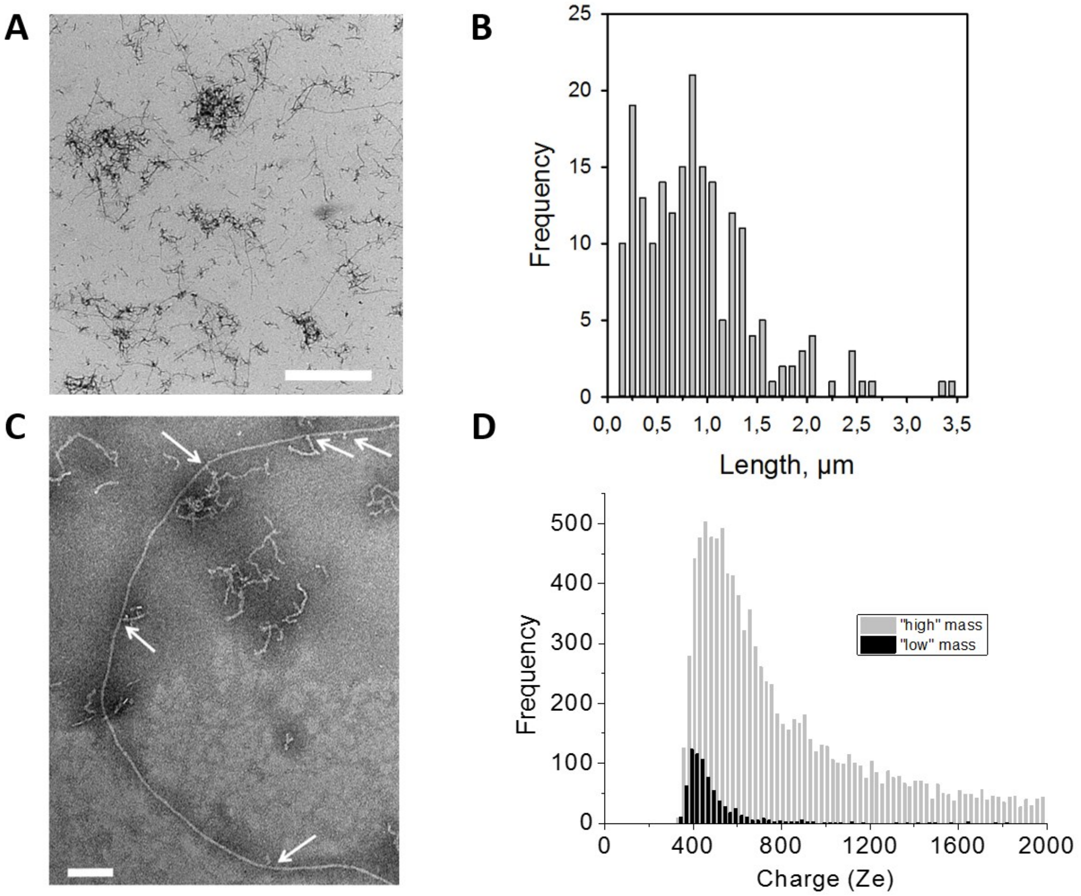

Figure S2: A) Low magnification TEM image of A$\beta_{1\text{-}42}$ amyloid fibers used for the length distribution; the protofibrils clusters were not considered for the length distribution. The white bar represents 2 µm. **B)** Length distribution of A$\beta_{1\text{-}42}$ amyloid fibers extracted from TEM images. The two types of fibrils were not distinguished for the length distribution. **C)** TEM image of A$\beta_{1\text{-}42}$ amyloid fibers, scale bar: 200 nm. The white arrows indicate branching points on the elongated fiber (see main text). **D)** Charge distribution drawn from Figure 2B. The two populations have been distinguished thanks to their different time of flight. The charge distribution is histogrammed using a given bin-size (25 e). Each bar represents the number of measured ions whose charges correspond to the charge range of the bin.

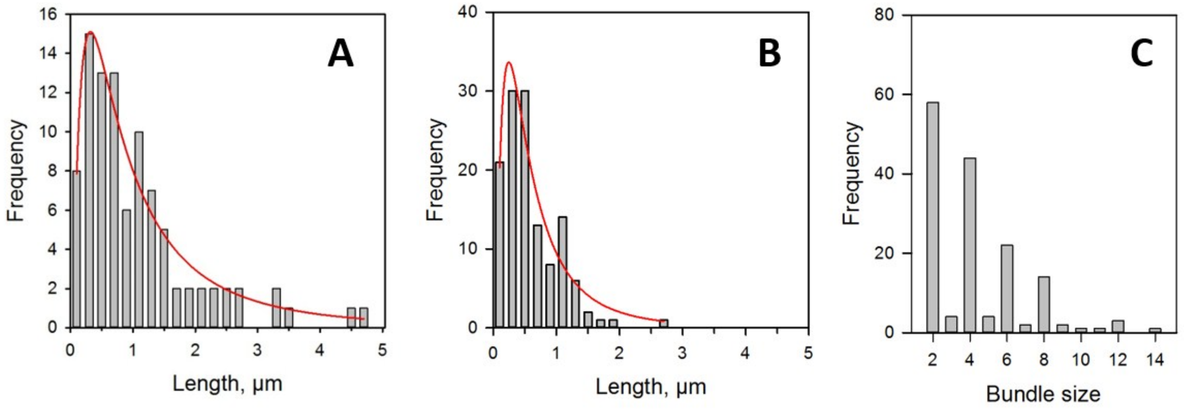

Figure S3: Length distribution of type I (**A)** and type II **(B)** α-synuclein amyloid fibers extracted from TEM images. The red curves represent the LogNormal function fitting the experimental distributions from which the mean length values were extracted (see main text). **(C)** Number of fibers involved in the ribbons. Along well defined ribbons, the number of fibers were determined every 300 nm; this is to overcome the heterogeneity of the ribbons.

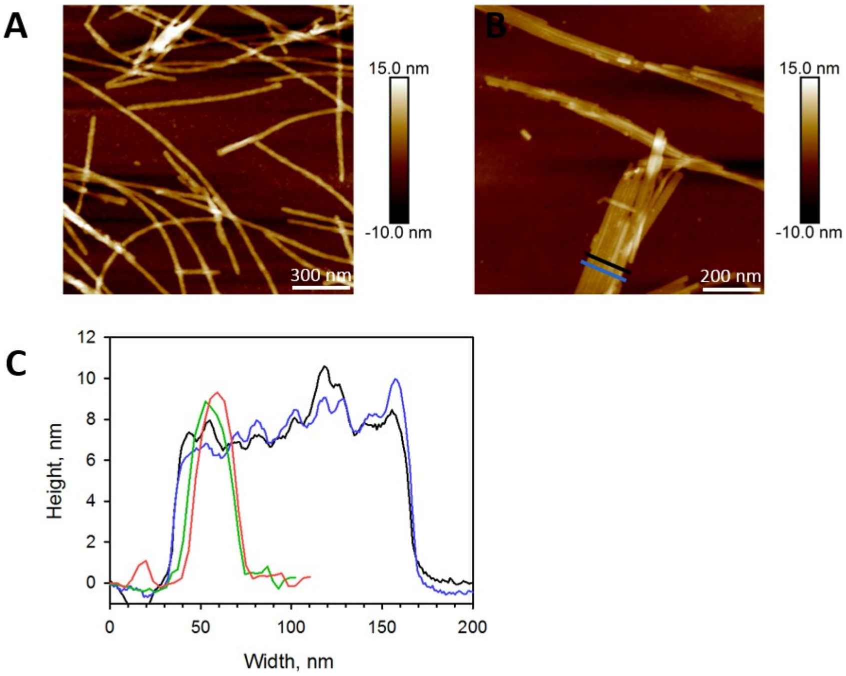

Figure S4: (A&B) AFM images of type I (**A)** and type II **(B)** α-synuclein amyloid fibers. **C)** Typical height profiles extracted from images shown on **A**) (green and red lines) and **B)** (black and blue lines).
